# Effect of Intracoronal Sealing Biomaterials on the Histological Outcome of Endodontic Revitalisation in Immature Sheep Teeth—A Pilot Study

**DOI:** 10.3390/jfb14040214

**Published:** 2023-04-11

**Authors:** Elanagai Rathinam, Sivaprakash Rajasekharan, Heidi Declercq, Christian Vanhove, Peter De Coster, Luc Martens

**Affiliations:** 1 ELOHA (Equal Lifelong Oral Health for All) Research Group, Paediatric Dentistry, Oral Health Sciences, Ghent University Hospital, 9000 Ghent, Belgium; 2 Tissue Engineering and Biomaterials Group, Department of Human Structure and Repair, Ghent University Hospital, Ghent University, 9000 Ghent, Belgium; 3 Tissue Engineering Laboratory, Department of Development and Regeneration, KU Leuven, 8500 Kortrijk, Belgium; 4 Medical Imaging & Signal Processing, Infinity Laboratory, Ghent University Hospital, Ghent University, 9000 Ghent, Belgium; 5 Department of Reconstructive Dentistry and Oral Biology, Dental School, Ghent University Hospital, Ghent University, 9000 Ghent, Belgium

**Keywords:** Biodentine, dental pulp, immature teeth, mineral trioxide aggregate, MTA, regenerative endodontics, revitalisation

## Abstract

The influence of intracoronal sealing biomaterials on the newly formed regenerative tissue after endodontic revitalisation therapy remains unexplored. The objective of this study was to compare the gene expression profiles of two different tricalcium silicate-based biomaterials alongside the histological outcomes of endodontic revitalisation therapy in immature sheep teeth. The messenger RNA expression of TGF-β, BMP2, BGLAP, VEGFA, WNT5A, MMP1, TNF-α and SMAD6 was evaluated after 1 day with qRT-PCR. For evaluation of histological outcomes, revitalisation therapy was performed using Biodentine (n = 4) or ProRoot white mineral trioxide aggregate (WMTA) (n = 4) in immature sheep according to the European Society of Endodontology position statement. After 6 months’ follow-up, one tooth in the Biodentine group was lost to avulsion. Histologically, extent of inflammation, presence or absence of tissue with cellularity and vascularity inside the pulp space, area of tissue with cellularity and vascularity, length of odontoblast lining attached to the dentinal wall, number and area of blood vessels and area of empty root canal space were measured by two independent investigators. All continuous data were subjected to statistical analysis using Wilcoxon matched-pairs signed rank test at a significance level of *p* < 0.05. Biodentine and ProRoot WMTA upregulated the genes responsible for odontoblast differentiation, mineralisation and angiogenesis. Biodentine induced the formation of a significantly larger area of neoformed tissue with cellularity, vascularity and increased length of odontoblast lining attached to the dentinal walls compared to ProRoot WMTA (*p* < 0.05), but future studies with larger sample size and adequate power as estimated by the results of this pilot study would confirm the effect of intracoronal sealing biomaterials on the histological outcome of endodontic revitalisation.

## 1. Introduction

Revitalisation therapy is based on the theory that undifferentiated stem cells and progenitor cells in the apical papilla of immature permanent teeth can be recruited in the root canal to regenerate functional dentine–pulp complex, induce deposition of mineralized tissue on the root canal wall and replace the necrotic pulp tissue lost due to caries, trauma or developmental anomalies [1,2]. Revitalisation therapy hypothesizes that in contrast to apexification therapy, apexogenesis will occur alongside the strengthening of the root canal walls and consequently prevent cervical fractures and increase the survival rate [3]. An important goal of revitalisation therapy is to form new tissue with the same histological characteristics to recapitulate the lost dental pulp. A majority of the animal and human studies, however, report the formation of pulp-like, cementum-like, bone-like, periodontal ligament-like or fibrous connective tissue [4].

The meticulous combination and interplay of three key elements, namely, stem cells, scaffold and bioactive molecules is referred to as the triad of tissue engineering [5,6]. The intentional manipulation of these three components of the tissue engineering triad has been investigated, but thus far, no protocol can achieve predictable endodontic tissue regeneration [7]. Stem/progenitor cells recruited from the apical region of the tooth are derived from peripheral blood, periodontal ligament, bone marrow or granulation tissue [8]. This has been proposed as a reason for the varied histology of the newly formed tissue [9]. Induced blood clot is the traditionally used scaffold, but certain studies suggest that blood clot might not be a stable scaffold to support tissue regeneration at its initial phase [10]. Alternative natural or synthetic scaffolds such as polylactic acid, polyglycolic acid, polycaprolactone, collagen, fibrin, chitosan, hyaluronic acid, poly(lactic-co-glycolic acid), peptide hydrogels, platelet-rich plasma and platelet-rich fibrin have been experimented with as possible scaffolds to enhance the histological outcome of revitalisation therapy [4,11].

Growth factors are critical signalling molecules that guide the stem cells towards differentiation [12]. These bioactive molecules are delivered at the site of regeneration from various sources such as demineralized dentine matrix fossilized with growth factors [13], paracrine/trophic effects of the stem cells [14,15], scaffolds impregnated with growth factors [16] and the molecular signalling induced with the biomaterial used as the intracoronal barrier. Tricalcium silicate cements (TSCs) are the most preferred biomaterials used for coronal seal during revitalisation therapy [17]. TSCs have been shown to induce the dental pulp stem cell differentiation regulated by a complex network of signalling molecules, pathways, receptors and transcription control systems [18,19].

Extensive literature is available on the variables that affect the histological outcome of revitalisation therapy such as age of the patient [20,21], diameter of open apex [21], extent of pulp necrosis [22,23], residual pulp in the canal [24], residual bacteria [25], disinfectants [26,27], irrigants [9,28,29], intracanal medicaments [28], stem cells [30] and scaffolds [4]. However, there are no studies comparing the influence of the intracoronal biomaterial on the histology of the newly formed regenerative tissue. Hence, the objective of this study was to compare the gene expression profiles of two different tricalcium silicate based biomaterials, namely, Biodentine and ProRoot white mineral trioxide aggregate (WMTA) alongside histological outcomes of revitalisation therapy in immature sheep teeth. The null hypothesis was that there is no difference in the histologic outcome between revitalisation therapy using Biodentine or ProRoot WMTA.

## 2. Materials and Methods

### 2.1. Cell Isolation

Human dental pulp stem cells (hDPSCs) were isolated from extracted unerupted human third molars with enzyme digestion [31]. Informed consent was collected from all patients, and ethical approval was obtained from the Ethical Committee of University hospital, Ghent, Belgium, according to the laws of the ICH Good Clinical Practice (GE11-LM-go-2006/57). The teeth were cleaned, cut with a bone cutter at the cemento–enamel junction to remove the pulp tissue and digested with type 1 collagenase and dispase. Small pieces of dental pulp tissue were transferred into enzyme solution for 1 h at 37 °C and vortexed every 30 min to break up the tissue. Thereafter, large cell aggregates were removed, and single-cell suspension was obtained via filtering through a cell-strainer (70 µM). The single-cell suspension was centrifuged at 1200 rpm for 5 min at room temperature. Supernatants were pipetted, and the pellet was resuspended in 1 mL basic medium (fetal bovine serum) to terminate enzymatic dissocation. Cell suspension was cultured in a 25 cm^2^ flask in Alpha-modified Eagles medium (α-MEM, Sigma-Aldrich, Overijse, Belgium) with 10% fetal bovine serum and antibiotics (100 U/mL penicillin and 100 mg/mL streptomycin) at 37 °C and 5% CO_2_. Then, culture medium was changed every 3 days until the cell confluency was achieved. Flow cytometry analysis was performed to identify the purity of the stem cell culture obtained. Purity of the custom prepared stem cells was determined as 96% using the mesenchymal stem cell markers CD90 and CD105.

### 2.2. Sample Preparation

ProRoot white MTA (WMTA) (Dentsply, Tulsa; Tulsa, OK, USA) and Biodentine (Septodont, Saint Maur des Fosses, France) were mixed according to the manufacturer’s instructions and allowed to set in Teflon moulds of 2 mm height and 8 mm diameter (n = 3 per group). The samples were allowed to set in 100% relative humidity at 37 °C for 3 h. The samples were removed from the mould and UV-sterilized for 30 min on each side. ProRoot white MTA and Biodentine were then placed in Transwell inserts of 0.4 µm in a 6-well plate seeded with hDPSCs at a density of 5 × 10^5^ cells/well and maintained at 37 °C and 5% CO_2_ for 1 day.

### 2.3. qRT-PCR Analysis

qRT-PCR analyses were completed after 1 day. cDNA was produced and amplified using a reverse transcriptome kit (QuantiTect Reverse Transcription kit, Qiagen, Hilden, Germany). Target cDNA was amplified using specific primer pairs. qRT-PCR was performed using the Sensimix SYBR No-ROX Kit (Bioline, London, UK) on a Light cycler 480 System (Roche Life Science, Penzberg, Germany). Samples were normalized using qBasePlus (Biogazelle NV, Zwijnaarde, Belgium) against at least three of the following genes: *Rpl13a*, *Eif4b*, *B2m*, *Actb*, or *Gapdh* as described previously [32]. Details of the specific primers used for gene expression analysis are provided in Table 1.

### 2.4. Animal Model

A double blind, split-mouth design, randomized controlled trial with sheep was designed and reported according to the ARRIVE (Animal Research: Reporting of In-vivo Experiments) and CONSORT (CONsolidated Standards of Reporting Trials) guidelines. The PICOT question was as follows: Did the sheep (P) receiving revitalisation therapy with Biodentine (I) in comparison to those receiving ProRoot WMTA (C) show a different histological outcome (O) after 6 months’ follow-up (T)? Due to lack of previous literature with quantitative histological data upon which sample size analysis could be calculated, we decided to perform a pilot study with post hoc sample size analysis. In accordance to a study by Altaii et al. [17] with a similar model (sheep), therapy (regenerative therapy with TCS cement) and protocol, an initial sample size of four teeth per treatment group was considered for this pilot study. The study protocol was approved by the ethical committee on animal experiments, Faculty of Medicine and Health Sciences, University Hospital, Ghent, Belgium (ECD 16/40). Four Suffolk sheep (2 males and 2 females) aged between 12 and 18 months with two newly erupted mandibular immature central incisors (two-tooth stage) were recruited. The sheep were housed in pairs in a stable with natural light during the treatment period. A preoperative digital occlusal radiograph (Dürr Dental Ag and VistaScan Perio, Bietigheim-Bissingen, Germany) was taken to ensure the presence of an immature open apex.

### 2.5. Randomization and Blinding

Simple randomization was performed for both the tooth and the biomaterial group depending on a set of randomized numbers generated with a computer using Matlab version 8.0 (https://nl.mathworks.com/products/matlab.html, accessed on 10 July 2021, The Mathworks Inc., Natick, MA, USA) software. The randomization list was concealed in a sealed envelope. The chosen tooth was randomly allocated by an independent individual (S.R) to one of the two groups: the Biodentine (Septodont, Saint Maur des Faussés, France) group or the ProRoot WMTA (Dentsply, Tulsa Dental, OK, USA) group. The other tooth was automatically allocated to the other group. All teeth were coded with an eight-digit code, where the first four digits were coded for the sheep number and the last four digits were coded for the tooth number and the biomaterial used. These unique codes ensured that the assessors (oral pathologists) were blinded to the type of treatment performed. A single precalibrated well-experienced operator (L.M) performed all the intervention and was blinded to the treatment group until the placement of the biomaterial.

### 2.6. General Anaesthesia

All procedures were performed under general anaesthesia. First, premedication (Domosedan, detomidine hydrochloride, 0.04 mg/kg intramuscular) was administered to the sheep. Induction of anaesthesia was implemented intravenously with midazolam 0.2 mg/kg and ketamine 6 mg/kg. Anaesthesia was maintained using 2% sevoflurane. Duratears, (Dextran 70, Hypromellose) was used for the protection of the cornea due to risk of dehydration.

### 2.7. Intervention

Endodontic revitalisation therapy was conducted according to the European Society of Endodontology (ESE) position statement on revitalisation procedures [33].

### 2.8. Phase 1

Coronal access was obtained lingually with a high-speed cylindrical diamond bur, and the pulp tissue was inoculated with a mixture of supragingival plaque and 2 mL of saline (Mini-Plasco, Braun, Diegem, Belgium). A cotton pellet soaked in the plaque suspension was placed on the exposed pulp tissue, and the access cavity was left open to induce necrosis of the pulp. All sheep were housed under the same conditions, fed in line with a natural diet and monitored for symptoms of postoperative pain for 4 weeks.

### 2.9. Phase 2

At each phase, the teeth were clinically examined for fractures, gingival swelling, abscess and/or fistula. Experimental teeth were isolated with a rubber dam, and the access cavity was reopened with a high-speed cylindrical diamond bur. Necrosis was confirmed clinically by the presence of necrotic pulp tissue and/or intracanal pus. At this stage, in all the sheep, a thin dentine bridge was observed both clinically and radiographically at the middle third of the root. The thin dentine bridge was mechanically removed with endodontic files. For reasons of anatomy, tooth orientation in the sheep mandible and facilitation of instrumentation, the access cavity was enlarged into the buccal area of the crown. After removal of all loose/necrotic pulp tissue, mild mechanical instrumentation of the root canal was performed. Root canals were irrigated with 20 mL of 3% sodium hypochlorite (NaOCl, Denteck, Zoetemeer, Belgium) using a side-vented needle. Bleeding or exudate within the root canal was controlled with paper points and irrigated with 5 mL of sterile physiological saline. After a drying with paper points (Dentsply, York, PA, USA), the root canal was finally irrigated with 20 mL of 17% ethylenediamenetetraacetic acid (EDTA, Denteck, Zoetemeer, Belgium). Calcium hydroxide paste (Ultracal, Ultradent, UT, USA) was injected in the root canal, a sterile cotton pellet was placed on the intracanal dressing and the cavity was sealed with glass-ionomer cement (Ketac Fill Aplicap; 3M ESPE, Seefeld, Germany). All sheep were housed under the same conditions, fed in line with a natural diet and monitored for symptoms of postoperative pain for 2 weeks. Discomfort in sheep was evaluated initially by means of monitoring behavioural changes such as pacing, agitation, vocalization and decreased appetite. Upon identification of behavioural changes, physiological parameters such as pupil dilatation and change in respiratory rate were investigated.

### 2.10. Phase 3

The access cavity was reopened with a high-speed cylindrical diamond bur, and the intracanal dressing was removed using irrigation with sterile physiological saline. After a drying with paper points, the root canal was irrigated with 20 mL of 17% EDTA followed by 5 mL of sterile physiological saline. After a drying with paper points, a sterile size-40 Hedström file (Dentsply, Washington, DC, USA) was used to mechanically irritate the apex in a rotational movement to induce bleeding into the root canal. The canal was allowed to fill with blood up to 2 mm below the gingival margin. Hemocollagene (Septodont, Saint Maur des Faussés, France) was placed at the coronal part of the root canal to a height of 2–3 mm on top of the blood clot.

**Figure 1:**
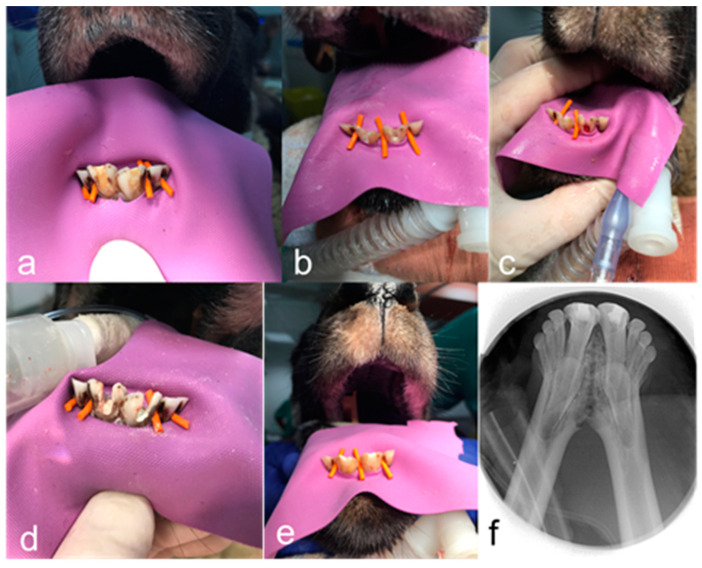
Representative clinical and radiographic images. (**a**) Preoperative image after rubber dam isolation of teeth 31 and 41. (**b**) Visualization of calcium hydroxide after access opening. (**c**) Induction of bleeding in the root canal of tooth 41. (**d**) Placement of intracoronal sealing material: Biodentine in tooth 31 and ProRoot White MTA in tooth 41. (**e**) Restorative build-up of teeth 31 and 41 after revitalisation therapy. (**f**) Postoperative radiographic image of teeth 31 and 41. Phase 4.

At this stage, the tooth was allocated to one of the intervention groups. In both the groups, the cement was mixed according to the manufacturer’s instructions, and a homogenous layer of about 2 mm was placed underneath the cemento–enamel junction. The tooth was restored with glass-ionomer cement (Ketac Fill Aplicap; 3M ESPE, Seefeld, Germany). The steps of phase 3 are shown in Figure 1. During the follow-up period of 6 months, the sheep were maintained and fed in their natural habitat.

A follow-up digital occlusal radiograph (Dürr Dental Ag and VistaScan Perio, Bietigheim-Bissingen, Germany) was taken, and the teeth were extracted. The extracted teeth were stored in 10% neutral-buffered formalin (PFA, VWR International, Radnor, PA, USA). After extraction of the teeth, the sheep were followed up for one week and sent to rehabilitation.

### 2.11. Radiographic Analysis

Quantitative radiographic analysis of intraoral radiographs was not possible, as the radiographs could not be standardized. Cone-beam µCT scans were acquired on a small animal radiation research platform (SARRP, XStrahl, Surrey, UK) [34]. A tube voltage of 90 kV was used in combination with a tube current of 200 µA and a 1 mm Al-filter. A total of 2000 projections were acquired over 360 degrees on a 20 × 20 cm flat-panel amorphous silicon detector with 1024 × 1024 pixels using a circular trajectory with continuous rotation and a magnification factor of 1.5. The acquired projection data were reconstructed into a three-dimensional DICOM image with a 512 × 512 × 512 matrix and a 75 µm isotropic voxel. Radiographic measurements of total root canal area and area of mineralized tissue were performed by setting binary thresholds using Digimizer software version 5.4.1 (https://www.digimizer.com, accessed on 10 July 2021, MedCalc Software, 8400 Ostend, Belgium) on 10 central slices per tooth. Percentage of mineralized tissue within the root canal was calculated as the ratio of the area of mineralized tissue to that of the total area of the root canal.

### 2.12. Histological Analysis

The teeth were decalcified in a combination of hydrochloric acid and EDTA (Decalcifier DC2 QPath, VWR International, Oud Heverlee, Belgium) and were embedded in paraffin wax after complete decalcification. Serial longitudinal sections 5 µm in thickness were cut and stained with haematoxylin and eosin (VWR International, Oud Heverlee, Belgium) for histological analysis. All slices were scanned with a microscope (Olympus BX51, Olympus, Tokyo, Japan) equipped with Xcellence software version 2.7 (https://www.olympus-lifescience.com/en, accessed on 10 July 2021, Olympus, Tokyo, Japan). All images were analysed independently by an oral pathologist (E.R) and an expert in oral biology (P.D.C).

Three continuous central sections with well-preserved tissue and absence of any artefacts were selected for histological analyses. For each section, 15 to 20 representative fields at 200× magnification covering the entire section were analysed. The following histopathological findings were evaluated using Digimizer software version 5.4.1 (https://www.digimizer.com, accessed on 10 July 2021, MedCalc Software, 8400 Ostend, Belgium).

1. Extent of inflammation was scored from 0 to 4 as follows: score 0, absent—absence of inflammatory cells; score 1, mild—small number of scattered inflammatory cells; score 2, moderate—some foci of inflammatory cells; score 3, severe—intense infiltration with inflammatory cells and altered tissue architecture; and score 4: necrosis: amorphous clumps of tissue remnants.
2. Presence or absence of tissue with cellularity and vascularity inside the pulp space was scored from 0 to 3 as follows: score 0—no tissue in-growth into the canal space; score 1—evidence of tissue in-growth into the apical third of the canal; score 2—evidence of tissue in-growth extending to the middle third of the canal; and score 3—evidence of tissue in-growth extending to the cervical third of the canal.
3. For area of tissue with cellularity and vascularity, only soft tissue with the presence of cells and blood vessels were measured.
4. Length of odontoblast lining attached to the dentinal wall was measured on both sides of the root for each tooth.
5. Number of blood vessels in each section was counted.
6. Area of blood vessels expressed as percentage of vascularity was calculated as the ratio of area of blood vessels to that of the area of tissue with cellularity and vascularity within each histological section.
7. The area of empty root canal space was measured as the area inside the root canal where neither soft nor hard tissue structures were present.

The ordinal scores for the first two parameters were adapted from previous studies by Tawfik et al. [35]. and Fahmy et al. [36]. The other parameters were measured on a continuous numerical scale to allow effect size and power analysis calculations.

### 2.13. Statistical Analysis

All measurements were performed by two independent evaluators (E.R and S.R), and any discrepancy in scoring was solved by a third person (P.DC). The evaluators repeated the measurements after two weeks. The interclass correlation coefficient and intraclass correlation coefficient were calculated using Statistical Package for Social Sciences (SPSS) v25.0 (IBM Corp., Armonk, NY, USA). All continuous data were subjected to statistical analysis with the Wilcoxon matched-pairs signed rank test at a significance level of *p* < 0.05 with GraphPad Prism version 6 (https://www.graphpad.com, accessed on 10 July 2021, GraphPad software Inc., San Diego, CA, USA). Effect size (Hedges’, g) was calculated for all continuous measurements. Cohen’s effect size index was used to classify the effect size as small (0.2), medium (0.5), large (0.8) and very large (1.3) [37]. GPower version 3.1 [38] (http://www.psychologie.hhu.de/arbeitsgruppen/allgemeine-psychologie-und-arbeitspsychologie/gpower.html, accessed on 10 July 2021) was used to perform calculations on sample size, effect size and statistical power. The minimal significance (α) and statistical power (1 − β) were set at 0.05 and 0.80, respectively.

## 3. Results

### 3.1. Gene Expression

**Figure 2:**
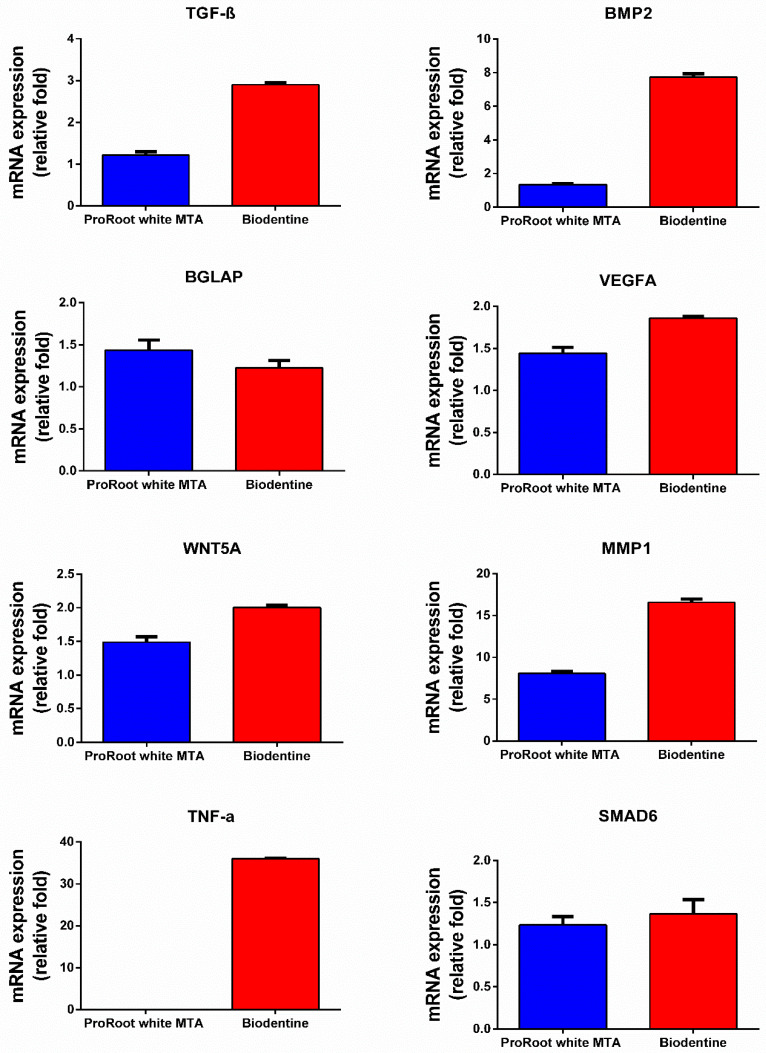
qRT-PCR expression. mRNA expression of TGF-β, BMP2, BGLAP, VEGFA, WNT5A, MMP1, TNF-α and SMAD6 after 1 day. The histogram shows upregulated mRNA expression in relative fold change. The details of the specific primers used for gene expression analysis are provided in Table 1.

Differential gene expression was observed between the two TCS-based biomaterials on eight specific gene markers (*TGF-β*, *BMP2*, *BGLAP*, *VEGFA*, *WNT5A*, *MMP1*, *TNF-α* and *SMAD6*) known to play a role in the mineralisation, angiogenesis and osteo/odontoblastic differentiation of stem cells (Figure 2). All genes were upregulated by both Biodentine and ProRoot WMTA, but *TNF-α* was not expressed by ProRoot WMTA. With the exception of *BGLAP*, a larger increase in mRNA expression (fold change) was observed with Biodentine compared to ProRoot WMTA after 1 day.

### 3.2. Qualitative Analysis of Revitalisation Therapy

None of the sheep had any discomfort after the revitalisation treatment. One sheep showed symptoms of pain between phase 2 and phase 3 (after the placement of the intracanal dressing). Buprenorphine 0.01 mg/kg was administered intramuscularly, and the pain subsided after three days. After phase 3 treatment, the animals were housed in their natural habitat for a period of 6 months. During this period, one sheep experienced an avulsion of one tooth (treated with Biodentine). The other tooth treated with ProRoot WMTA in the same sheep was intact and was included in the analysis. No other adverse events were observed during the trial. The radiographic outcomes were in accordance with the histological analyses.

**Figure 3:**
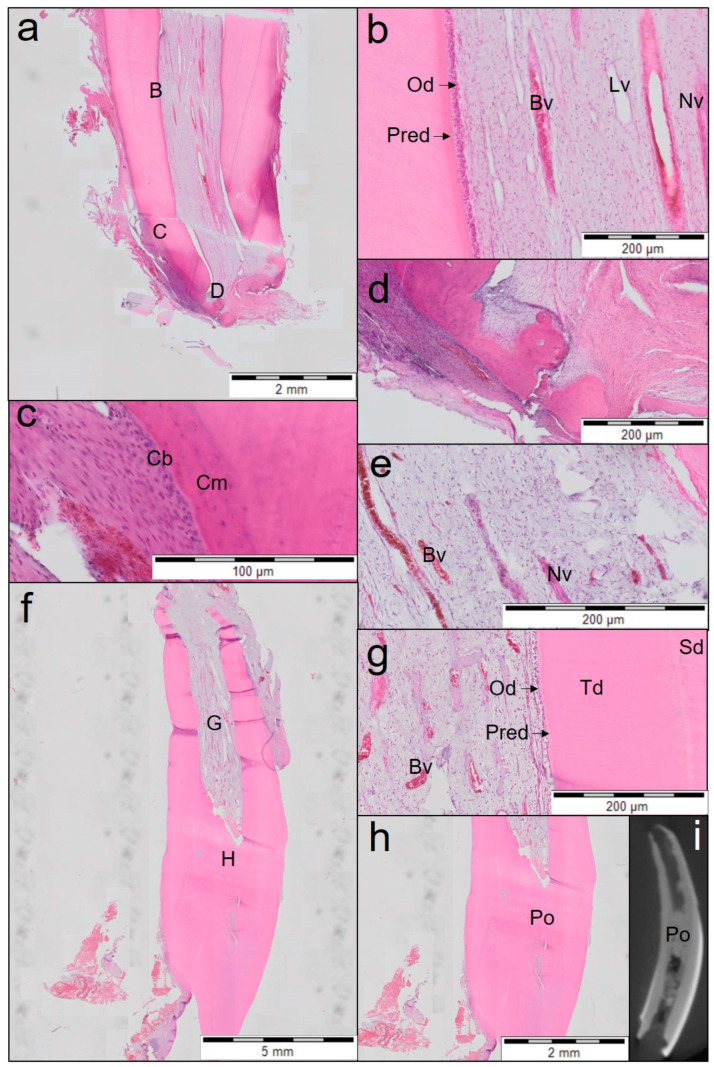
Overview of the first and second teeth treated with Biodentine (haematoxylin and eosin staining; (**a**–**e**) the first tooth; (**f**–**i**) the second tooth). (**a**) Midsagittal section of the middle and the apical part of the root (first tooth). Individual letters (B, C, D) represent the regions of interest. (**b**) Magnified view of region B from (**a**) shows odontoblast (Od) lining attached to the dentinal walls and neoproliferation of blood vessels (Bv) with a dense amount of collagen fibres which is composed of a large number of fibroblasts maintained throughout the middle and apical third of the root. Predentine (Pred), lymph vessels (Lv) and nerves (Nv) can also be seen in this section. (**c**) Magnified view of the region C from (**a**) shows cementum (Cm) with a lining of cementoblast-like cells (Cb). (**d**) Magnified view of the apical region D from (**a**) showing cellular cementum on both sides with narrowing of the apical foramen. (**e**) Section of the first tooth showing nerves (Nv) and blood vessels (Bv) in the middle third of the root. (**f**) Midsagittal section of the coronal, middle and apical third of the root (2nd tooth). Individual alphabets (G, H) represent regions of interest. (**g**) Magnified view of the region G from (**f**) showing reorganized pulp-like tissue in the middle and apical third of the tooth. Excessive neoproliferation of blood vessels (Bv) and restructured pulp-like tissue with odontoblast-like cells lining the dentinal walls. Secondary dentine (Sd), tertiary dentine (Td) and predentine (Pred) with odontoblast (Od) lining. (**h**) Magnified view of the coronal third of the pulp (region H from (**f**)) showing coronal pulp obliteration (Po). (**i**) µCT image showing coronal pulp obliteration (Po).

Teeth treated with Biodentine (n = 3) presented 2 distinctive histological patterns of the newly regenerated tissue. Two of the three teeth treated with Biodentine (Figure 3) showed clear deposition of tertiary dentine attached to the dentinal walls resulting in root wall thickening. The apices showed lengthening of the root and narrowing by cementum-like tissue. Periodontal ligaments surrounding the teeth were dense and vascular connective tissue was seen. Middle and apical root thirds showed signs of pulp vitality with odontoblast-like cells lining the root canal. The canal showed a rich neoproliferation of blood vessels, nerves and other cells such as fibroblasts. In one of these teeth, obliteration at the coronal third of the pulp was observed.

**Figure 4:**
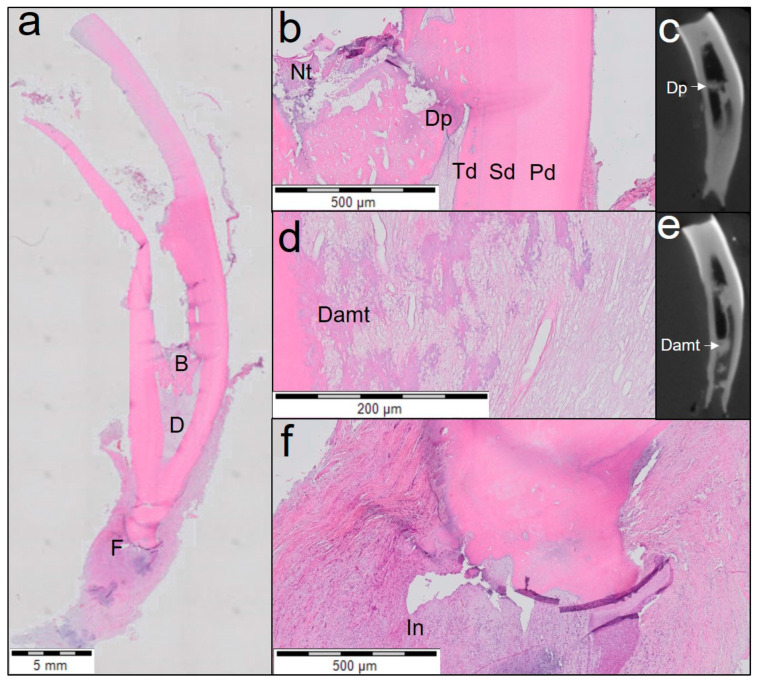
Overview of the third tooth treated with Biodentine (haematoxylin and eosin staining). (**a**) Midsagittal section of coronal, middle and apical third of the root (3rd tooth). Individual letters (B, D, F) represent regions of interest. (**b**) Magnified view of region B from (**a**) shows three distinguished layers of dentine (primary dentine (Pd), secondary dentine (Sd) and tertiary dentine (Td) with a dentine plug (Dp) separating the necrotic tissue (Nt). (**c**) A µCT image showing the dentine plug (Dp). (**d**) Magnified view of region D from (**a**) shows dentine-associated mineralisation (Damt) with fibrous connective tissue. (**e**) A µCT image showing dentine-associated mineralisation tissue (Damt). (**f**) Magnified view of the periapical region (region F from (**a**)) showing chronic inflammation with rich inflammatory infiltration (In) and apex closure.

One tooth treated with Biodentine (Figure 4) showed closing of the apex by cementum-like tissue even though periapical inflammation was present. This tooth also showed a distinguished layer of tertiary dentine attached to the dentinal walls and a dentinal plug separating vital from necrotic pulp tissue. The middle and the apical third of the canal showed fibrous connective tissue with poor cellularity.

**Figure 5:**
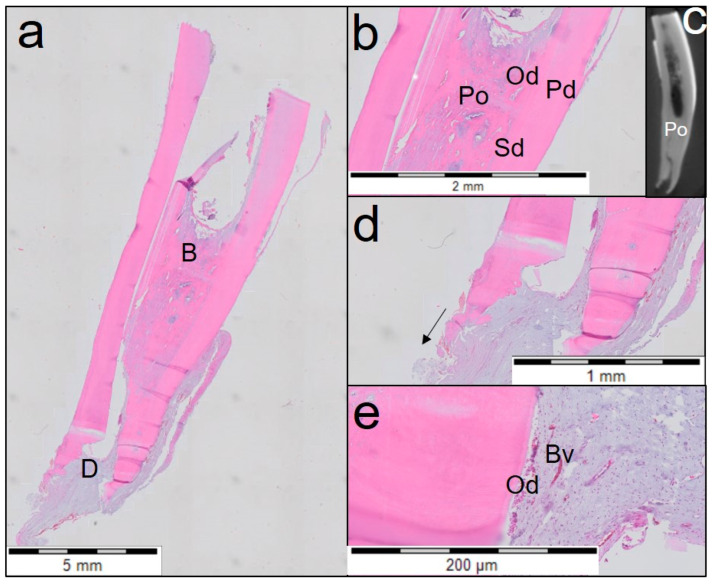
Overview of the first tooth treated with ProRoot WMTA (haematoxylin and eosin staining). (**a**) Midsagittal section of the coronal, middle and apical third of the root (first tooth). Individual letters (B, D) represent regions of interest. (**b**) Magnified view of region B from (**a**) shows pulp obliteration (Po) by osteodentine (Od), and the dentinal wall on the right shows complete void throughout the canal. Primary dentine (Pd) and secondary dentine (Sd) can also be seen in this section. (**c**) A µCT image showing pulp obliteration (Po). (**d**) Magnified view of region D from (**a**) shows lengthening of the apical third of the root (indicated by an arrow). (**e**) Magnified view of the apical third of the root showing few areas with discontinuous lining of odontoblast-like cells (Od) and blood vessels (Bv). Fibrovascular connective tissue with poor cellularity is seen.

Teeth treated with ProRoot WMTA also presented two distinctive histological patterns of the newly regenerated tissue. One tooth treated with ProRoot WMTA (Figure 5) presented with a distinguished layer of tertiary dentine which was attached to the dentinal walls on only one side and detached on the other side. Lengthening of the roots with cementum-like tissue and a discontinuous lining of odontoblast-like cells in the apical third of the root were seen. This was observed only unilaterally; very few odontoblast-like cells were seen at the contralateral side. The middle and the coronal third of the roots were obliterated with osteodentine-like structures with dispersed cellular inclusions. The middle and the apical third of the canal spaces contained fibrovascular connective tissue with poor cellularity.

**Figure 6:**
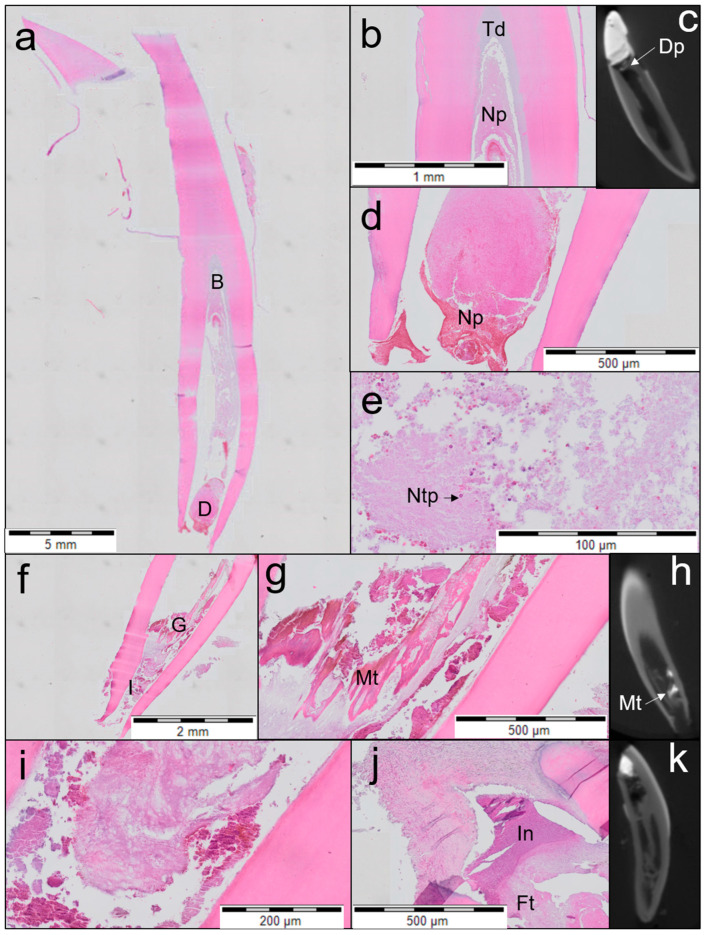
Overview of the second, third and fourth teeth treated with ProRoot WMTA (haematoxylin and eosin staining; (**a**–**e**) second tooth; (**f**–**i**) third tooth; (**j**,**k**) fourth tooth). (**a**) Parasagittal section of the coronal, middle and apical third of the root (secibd tooth). Individual letters (B, D) represent regions of interest. (**b**) Magnified view of region B from (**a**) shows moderate formation of tertiary dentine (Td) and necrotic pulp (Np) in the root canal. (**c**) A µCT image showing formation of a dentine plug (Dp) not attached to the dentine walls on the left side (**d**) Magnified view of region D from (**a**) showing the apical third of the canal with open apex, necrotic pulp (Np) with aggregation of red blood cells and few inflammatory cells. (**e**) Magnified view of the middle third of the root shows necrotic tissue with moderate infiltration of neutrophils (Ntp). (**f**) Midsagittal section of the apical and middle third of the root (3rd tooth). Individual letters (G, I) represent regions of interest. (**g**) Magnified view of region G from (**f**) shows mineralized tissue (Mt) not attached to the dentinal wall. (**h**) A µCT image showing mineralized tissue (Mt). (**i**) Magnified view of region I from (**f**) showing loosely arranged fibrotic tissue filling the apical and middle third of the root, with blood cells and few residual inflammatory cells indicating incomplete recovery from inflammation. (**j**) Inflammatory infiltration (In) in the apical third of the root canal and infection with an open apex. Fibrotic tissue (Ft) can be seen between the apical third and the middle third of the root with few inflammatory cells. (**k**) A µCT image showing fibrotic tissue in the root canal.

In three of the four teeth treated with ProRoot WMTA (Figure 6), no distinguished layer of tertiary dentine attached to the dentinal walls was observed, and the root walls were remarkably thin. The apical and middle thirds of the root canals were filled with loose, sparse fibrotic tissue with areas showing aggregates of red blood cells and inflammatory cells. No prominent narrowing of the root apices was observed. In one tooth, an inflammatory cell infiltrate was observed at the apical region.

### 3.3. Quantitative Analysis of Revitalisation Therapy

The interclass correlation coefficient between the evaluators was excellent (0.961; CI 0.888–0.987). The intraclass correlation coefficient was also excellent: 0.984 (CI 0.943–0.996) for evaluator 1 (E.R) and 0.98 (CI 0.941–0.993) for evaluator 2 (S.R). The results of the histological and radiographic analyses are summarized in Table 2. Significantly more neo-formed intracanal tissue with cellularity and vascularity was seen in the Biodentine group compared to the ProRoot WMTA group (*p* < 0.05). Likewise, the length of odontoblast lining attached to the dentinal wall was significantly increased in teeth treated with Biodentine compared to those treated with ProRoot WMTA (*p* < 0.05). No significant difference between the treatment groups were observed with the other parameters measured.

Hedges’ g is a measure of effect size weighted according to the relative size of each sample. It was used in the present study due to the discrepancy in sample size between the Biodentine and ProRoot WMTA groups, as one tooth in the Biodentine group was lost from avulsion during follow-up. In correlation with the significant results obtained, effect size could be categorized as very large for both areas of tissue with cellularity and vascularity (1.92) and length of odontoblast lining attached to the dentinal wall (1.86). For the number of blood vessels and percentage of vascularity, the effect size was large (1.46 and 1.27, respectively), but power analysis revealed inadequate power (0.26 and 0.32, respectively). Post hoc power analysis estimated that a sample size of a minimum of six teeth per group was necessary to achieve a power of 0.80. The effect size for the area of empty root canal space was medium (0.69), resulting in inadequate power (0.12), and a sample size no smaller than 36 teeth per group would be required to achieve a power of 0.80. The percentage of mineralized tissue within the root canal showed a small effect size (0.31) with inadequate power (0.06). A large sample size of at least 169 teeth per group would be required to achieve an adequate power (0.80) to show significant difference in the percentage of mineralized tissue.

The columns are colour coded such that the columns with same colour indicate the teeth from a single sheep. The data for histological quantification is calculated as the average from n = 3 sections for each tooth and from n = 10 slices for radiographic data. SEM values represent the standard error of the mean. Extent of inflammation was scored from 0–4. Score 0: Absent: Absence of inflammatory cells; Score 1: Mild: Small number of scattered inflammatory cells; Score 2: Moderate: Some foci of inflammatory cells; Score 3: Severe: Intense infiltration with inflammatory cells and altered tissue architecture; Score 4: Necrosis: Amorphous clumps of tissue remnants. Presence or absence of tissue with cellularity and vascularity inside the pulp space was scored from 0–3. Score 0: No tissue in-growth into the canal space; Score 1: Evidence of tissue in-growth into the apical third of the canal; Score 2: Evidence of tissue in-growth extending to the middle third of the canal; Score 3: Evidence of tissue in-growth extending to the cervical third of the canal. Area of blood vessels expressed as percentage of vascularity was calculated as the ratio of area of blood vessels to that of the area of tissue with cellularity and vascularity within each histological section. Area of mineralized tissue was expressed in percentage calculated as the ratio of area of mineralization to that of the total area of the root canal.

## 4. Discussion

Tri- and dicalcium silicate, tricalcium aluminate, calcium sulfate dihydrate and bismuth oxide constitute the powder component of ProRoot WMTA. On the other hand, the powder component of Biodentine is composed of tricalcium silicate, calcium carbonate, calcium oxide, iron oxide and zirconium oxide. The powder component of ProRoot WMTA is mixed with distilled water, but Biodentine is mixed with a liquid containing hydrosoluble polymer and calcium chloride to accelerate the setting time [39]. Although ProRoot WMTA and Biodentine are classified as TCS-based hydraulic cements, there is difference in the nature of synthesis, additives added, size of the particles and the composition of liquid medium [40]. These differences in composition is reflected in the difference in physical (compressive strength, push-out bond strength, density and porosity), biologic (immediate formation of calcium hydroxide, higher release and depth of incorporation of calcium ions) and clinical properties (handling and tooth discoloration) [41].

For studying dental pulp revitalisation outcomes, diphyodont animals such as ferret, rabbit, dog, sheep and primates with immature single-rooted permanent teeth are the model of choice [42]. Recent literature supports the use of sheep in the two-tooth stage as an appropriate animal model for revitalisation research due to their anatomic similarity to human teeth [17,43]. A split-mouth design was devised to eliminate the intersheep variability in terms of age of the sheep, developmental stage of the tooth and individual immune response from affecting the outcome of revitalisation therapy. In the current protocol, 3% NaOCl was chosen for disinfection and dissolution of pulp tissue remnants to limit the toxicity towards stem cells [44]. The concentration of NaOCl recommended by the ESE and American Association of Endodontologists (AAE) guidelines for revitalisation lies in the range of 1.5–3% to achieve the balance between sufficient disinfection and tissue preservation. EDTA was used, as it is the preferred choice of irrigant in the revitalisation protocol based on its ability to inhibit biofilm formation and demineralize the dentine to release growth factors sequestered into the calcified dentine matrix [5,45]. Calcium hydroxide was the intracanal medicament of choice, as antibiotic pastes can cause discoloration, sensitization, resistance and difficulty of removal from the root canal [46,47]. Both the ESE and AAE guidelines for revitalisation recommend calcium hydroxide as the first choice for disinfection, and the use of antibiotic pastes is recommended only in cases of persistent infection. Moreover, calcium hydroxide can prompt mineralisation due to its ability to solubilize dentine extracellular matrix and release growth factors [48]. Hemocollagene was verified to be beneficial for the placement of the TSCs at their optimum level without apical displacement or distortion of the newly formed blood clot [49].

In this study, blood clot was used as a scaffold. It stimulates the proliferation and differentiation of fibroblasts, odontoblasts and cementoblasts from their undifferentiated mesenchymal stem cells via platelet-derived growth factor, vascular endothelial growth factor and other tissue growth factors present in the blood clot. However, the disadvantages of using blood clot as a scaffold may include the uncertain composition, unknown breakdown kinetics, inability to have adequate bleeding in all cases and the necessity to traumatize apical tissues for evoking bleeding [50].

During the placement of intracanal medication (phase 2), a dentine bridge was clinically and radiographically observed in all teeth in the middle third of the roots. The inoculation with supragingival plaque (phase 1) mimicked the advancing infection found in deep (dentinal) caries lesions, where an acute immune reaction is triggered resulting in the formation of a dentine bridge [51]. During phase 2 pulpectomy, the pulp tissue proximal to the dentine bridges was clinically found to be necrotic, indicating that the initial immune reaction had failed to stop the propagation of local necrosis started at the invasion front. Previous authors indicated that, amongst others, the size of pulp exposure and the presence of infection are critical elements in success or failure of pulp repair [52,53].

Galler et al. characterized regeneration seen following revitalisation therapy as the restoration of pulp tissue architecture and function. Histologically, pulp-like tissue formation in root canals is observed where stem cells differentiate into odontoblasts. Conversely, repair has been defined as ectopic tissue formation with partial loss of function and formation of fibrous tissue, cementum or bone inside the root canal [33]. If this categorization was to the results of the present study, two of the three teeth treated with Biodentine showed regeneration while one tooth exhibited repair. On the other hand, histological outcomes of the ProRoot WMTA group revealed regeneration in one tooth and repair in three of the four treated teeth. However, the intracoronal sealing materials revealed significantly different histological outcomes; clinically and radiographically, all treated teeth were asymptomatic. By following the ESE guidelines for revitalisation of immature carious necrotic teeth, clinical and radiographic success was possible, while the histological outcome was unpredictable and nonreproducible.

A majority of the previous literature on regenerative endodontics fails to associate newly formed calcified tissues in the pulp space with the presence of odontoblasts [17]. Differentiation of stem cells into odontoblasts is a prerequisite for the formation of pulp-like tissue in root canals [54]. Biodentine and ProRoot WMTA are known to activate extracellular signal-regulated kinase ½, nuclear factor E2-related factor 2, p38, c-Jun N-terminal kinase mitogen-activated protein kinase, p42/p44 mitogen-activated protein kinase, nuclear factor kappa B and fibroblast growth factor receptor pathways to stimulate the osteogenic/odontogenic capacity of dental pulp stem cells via proliferation, angiogenesis and biomineralisation. The upregulated expression of *BMP2* indicates the ability of these cements to induce differentiation of dental pulp stem/progenitor cells into odontoblasts [55]. Our results show that Biodentine induced a fourfold higher expression of BMP2 compared to ProRoot WMTA. The upregulation of *SMAD6* limits *BMP* signalling for proper odontoblastic differentiation, as *SMAD6* provides feedback inhibition of BMP-receptor activation [56]. *BGLAP* is an osteoblast marker and is also considered to be a late differentiation marker of odontoblasts [57]. Both Biodentine and ProRoot WMTA upregulated the expression of *SMAD6* and *BGLAP*. In the present study, two teeth treated with Biodentine showed signs of a vital pulp with odontoblast-like cells lining the middle and apical third of the canal. In one of the teeth treated with ProRoot WMTA, the root canal was continuous with the periodontal ligament indicating a lack of closure of the apex. In these teeth, odontoblast-like cells were seen in a few zones which may hint at a probability of complete pulpal regeneration in the future.

Two teeth in Biodentine group presented with rich vascularity, lymphatic vessels and few nerves which were absent in the ProRoot WMTA group. Despite the higher number of blood vessels and increased vascularity percentage of Biodentine compared to ProRoot WMTA, no significant differences could be seen between the two groups due to low sample size of this pilot study. Previous authors have corroborated that dental pulp stem cells produce angiogenic and neurotropic factors for revascularization and reinnervation of the regenerated pulp [58,59,60]. This is in correlation with the results of other studies where the regenerated pulp was found to have high vascularity [61,62,63,64] and innervated with newly regenerated nerve fibres [9,61,62]. *VEGF* and *MMP1* are known to promote blood vessel formation-enhancing neovascularization in vivo [65]. Both these genes were upregulated by Biodentine and ProRoot WMTA after 1 day.

Lengthening of the root with cementum-like tissue and three distinguished layers of dentine was seen in all Biodentine-treated teeth and one tooth treated with ProRoot WMTA. Newly deposited dentine-like tissue with entrapped cells inside the calcified matrix was a finding similar to that of previous studies [66,67]. The deposition of tertiary dentine by odontoblasts suggests the differentiation of migrant stem cells elicited by transforming growth factor (*TGF-β*) after revitalisation therapy [68,69]. In the present study, Biodentine showed a higher expression of *TGF-β* than did ProRoot WMTA. Biodentine has been shown to be responsible for early reparative dentine synthesis by inducing *TGF-β1* release from dental pulp stem cells [70]. Similar results with lengthening of root by cementum-like tissue were reported earlier in immature sheep teeth treated with MTA [17]. Outward flow of dentinal fluids following pulp necrosis may cause odontoblast entrapment resulting in tubular or vacuolated reparative dentine [71].

Pulp injury causes secretion of neuropeptides by sensory nerves resulting in neurogenic inflammation. The pulp tissue becomes edematous with a rise in blood and interstitial fluid within the root canal causing the compression of thin-walled venules and increased resistance to blood flow. Decrease in blood flow causes aggregation of red blood cells and the elevation of blood viscosity inducing hypoxia or ischemia, suppressing cellular metabolism in the affected area and finally necrosis of the pulp [71]. This could explain the presence of red blood cells in three of the four ProRoot WMTA-treated teeth showing sparse fibrotic tissue in the canal space and no prominent narrowing of the root apex.

The ability of Biodentine and ProRoot WMTA to upregulate mineralisation-related genes [18], thereby inducing mineralisation and reparative dentine formation [72] has broadened the clinical indications of these materials to direct/indirect pulp capping [73], pulpotomy [74], furcation repair [75,76] and the treatment of resorption [77]. Other authors already have reported that reparative dentine formed by Biodentine in pulp capping studies is usually faster and thicker than are the ProRoot WMTA homologues [78,79,80,81]. Our results showed that both Biodentine and ProRoot WMTA upregulated the expression of *WNT5A*, but only Biodentine induced *TNF-α* expression. *WNT5A* and *TNF-α* induce mineralisation and mineralisation-related gene expression through nuclear factor kappa (NF-κB) signalling pathway in hDPSCs [82,83].

Although both Biodentine and ProRoot WMTA are tricalcium silicate-based cements, there existed marked differences in the gene expression profiles of these cements which could be attributed to the difference in purity, composition, particle size, mechanical properties and kinetics of calcium release [40]. Therefore, we hypothesize that the difference in gene expression profiles could be responsible for the different histological outcomes observed in the present study.

The limited number of teeth treated in each group prevented this pilot study from having adequate power (1 − β > 0.80) and effect size to determine a significant difference in vascularity (number of blood vessels and relative area). Post hoc power analysis showed that the low effect size demanded a very large sample size for area of empty root canal space (n = 36 teeth) and percentage of mineralized tissue in the root canal (n = 169 teeth). However, we believe that these are secondary parameters, as the primary goal of revitalisation therapy is to achieve pulp tissue architecture and function. Thus, the presence of neoformed soft tissue with blood vessels and an odontoblast lining attached to the dentinal wall should be considered as the primary characteristics for evaluating success. Based on the above assumption and taking into account the practical as well as the ethical aspects of conducting an animal study, we recommend that a sample size of a minimum of six teeth per group will be required to achieve adequate power (1 − β > 0.80) in future studies.

### 4.1. Limitations of the Present Study

Quantitative radiographic analysis of intraoral radiographs was not possible, as they could not be standardized. Superimposition of radiographs was not feasible owing to the dynamic alterations in the dentition of the sheep, namely, change in coronal height of erupting incisors, continuous attrition of the molars and the shedding of deciduous laterals/canines during the follow-up period. Extending the follow-up period was not possible in the present study due to the high cost of sheep maintenance. The histological outcomes of the present study are a simulation of revitalisation therapy after pulp necrosis subsequent to bacterial exposure, but the same may not be true for trauma or longstanding periapical infection due to possible changes in the viability of Hertwig’s epithelial root sheath. The difference in biological dynamics between sheep and humans should be considered before the extrapolation of the current results to humans. Although significant differences between the groups exist for some of the measured histological characteristics, the results need to be interpreted with much caution considering the low sample size of the pilot study. Future studies with larger sample sizes and adequate power as estimated by the results of this pilot study would yield more valuable and concrete information regarding the role of intracoronal sealing biomaterial on the histological outcome of endodontic revitalisation.

### 4.2. Conclusions

Biodentine induced the formation of a significantly larger area of neoformed tissue with cellularity, vascularity and increased length of odontoblast lining attached to the dentine walls compared to ProRoot WMTA (*p* < 0.05). Hence, the null hypothesis that there is no difference in the histologic outcome between revitalisation therapy using Biodentine or ProRoot WMTA is rejected. The intracoronal sealing material appears to play an ancillary role in the type of tissue formed in the root canal after revitalisation therapy, possibly through an interplay between signalling molecules, responsive cell populations and the microenvironment.

## Figures and Tables

**Table 1:** Details of the primers used in this study.

Target Gene	Primer	
	Forward	Reverse
***GAPDH***	CTACCAGTGCAAAGAGCCCA	TGGTCATCAACCCTTCCACG
***ACTB***	CTTCGCGGGCGACGAT	CCACATAGGAATCCTTCTGACC
***B2M***	ACTTAGAGGTGGGGAGCAGA	GCCCTTTACACTGTGAGCC
***EIF4b***	GTGCGTTTACCACGTGAACC	CGTGCATCCTGGTCTGACTT
***RPH3a***	CTGGTCCGAGTTTTCTCCGC	TTCTTTATCATTTGATTGAAGGGGC
***TGF-β1***	AGGGCTACCATGCCAACTTC	GACACAGAGATCCGCAGTCC
***BMP2***	AGTCCTGATGAGCATGAGCC	CTCACCTATCTGTATACTGC
***BGLAP***	CTCACACTCCTCGCCCTAT	TCTCTTCACTACCTCGCTGC
***VEGFA***	ATGCGGATCAAACCTCACCA	CACCAACGTACACGCTCCAG
***WNT5a***	AAGCAGACGTTTCGGCTACA	TTTCCAACGTCCATCAGCGA
***MMP1***	CCCAGCGACTCTAGAAACACA	CTGCTTGACCCTCAGAGACC
***TNF-α***	GTGACAAGCCTGTAGCCCAT	CTCTGATGGCACCACCAACT
***SMAD6***	AAAACCGTCACGTACTCGCT	GGTCGTACACCGCATAGAGG

**Table 2:** Quantification of histological and radiographic data.

	Biodentine						ProRoot WMTA							
	Tooth 1		Tooth 2		Tooth 3		Tooth 1		Tooth 2		Tooth 3		Tooth 4	
	Mean	SEM	Mean	SEM	Mean	SEM	Mean	SEM	Mean	SEM	Mean	SEM	Mean	SEM
Extent of inflammation (0–4)	**0**	0	**0**	0	**0**	0	**2**	0	**3**	0	**0**	0	**3**	0
Presence or absence of tissue with cellularity and vascularity inside the pulp space (0–3)	**2**	0	**2**	0	**0**	0	**0**	0	**0**	0	**1**	0	**0**	0
Area of tissue with cellularity and vascularity (mm^2^)	**13.14**	1.6	**11.78**	1.47	**0**	0	**0**	0	**0**	0	**0.23**	0.12	**0**	0
Length of odontoblast lining (mm)	**13.4**	1.29	**11.27**	1.43	**0**	0	**0**	0	**0**	0	**1.41**	0.72	**0**	0
Number of blood vessels (n)	**64.33**	19.33	**176.33**	19.62	**0**	0	**0**	0	**0**	0	**45**	16.77	**0**	0
Area of blood vessels expressed as percentage of vascularity (%)	**7.45**	1.85	**10.03**	0.99	**0**	0	**0**	0	**0**	0	**3.76**	0.16	**0**	0
Area of empty root canal space (mm^2^)	**1.36**	0.51	**1.15**	0.38	**23.70**	1.08	**11.69**	0.37	**12.91**	2.71	**11.03**	0.71	**22.28**	2.32
Area of mineralized tissue (%)	**0.33**	0.01	**0.26**	0.01	**0.28**	0.02	**0.27**	0.01	**0.08**	0.01	**0.38**	0.02	**0.31**	0.01

## Data Availability

The data presented in this study are available on request from the corresponding author. The data are not publicly available as this study is part of an ongoing project and only the result sof the pilot study are presented here.
